# Influencing Factors of the Body Mass Index of Elementary Students in Southern Taiwan

**DOI:** 10.3390/ijerph14030220

**Published:** 2017-02-23

**Authors:** Li-Na Chou, Min-Li Chen

**Affiliations:** 1 Department of Nursing, National Tainan Junior College of Nursing, No. 78, Min-Tsu Rd. Sec. 2, Tainan 700, Taiwan; lnchou@ntin.edu.tw; 2 Department of Respiratory Care, Graduate Institute of Nursing, Chang Gung University of Science and Technology, Chiayi Campus, West Sec., Jiapu Rd., Puzi City 61363, Chiayi County, Taiwan

**Keywords:** school children, body mass index (BMI), healthy body weight, health promotion

## Abstract

The body mass index (BMI) of school children in Taiwan is markedly increasing. According to statistical data from the Taiwan Ministry of Education, the prevalence of obesity in school children from the southern part of the country is the highest in Taiwan. Thus, exploring the factors influencing BMI in elementary school children from southern Taiwan is crucial. This study investigated the influencing factors including physical activity levels, sedentary behaviors, dietary habits, and perceived body shape on the BMIs of elementary school children from southern Taiwan. A cross-sectional design was used, and the participants consisted of 3251 fifth-grade students (1628 boys, 50.1%; 1623 girls, 49.9%). The average BMI values for boys and girls were 19.69 and 18.70 (kg/cm) respectively. Statistically significant associations were observed between BMI and sex, 31–60 min of daily vigorous or moderate physical activities levels, length of time spent watching television, time spent on video games or the computer, and intake of vegetable or meat gravy with rice (*p* < 0.001). Perceived body shape also affected the BMI of school children. The results of this study enable educational institutions in Taiwan to understand the factors affecting the BMI of school children and use this information as the basis for future healthy body weight policies.

## 1. Introduction

Over the past 10 years, the prevalence of childhood obesity has been on the rise and has become a significant public health concern. In 2014, an estimated 41 million children under the age of 5 years were overweight or obese [1]. The body mass index (BMI) of children and adolescents in Taiwan has also displayed a significant annual upward trend. Among the 28 countries of the International Obesity Taskforce (IOTF), Taiwan is ranked seventh, alongside the United States, in terms of childhood overweight (and obesity) prevalence; this prevalence is much higher than that of major European countries and approximately twice that of Mainland China [2]. The rate of obesity in school children in Taiwan has reached 29.72%, indicating that three in 10 school children are obese. This situation is especially dire in the southern counties. A report released by the Taiwan Ministry of Health and Welfare in 2011 revealed that the southern county had the highest rate of obesity in the country [3]. In 1996, the WHO classified obesity as a chronic disease, and noted that the global incidence of chronic diseases and mortality rates resulting from obesity appeared to be increasing annually [4]. Obesity has physical and psychological health consequences during childhood, adolescence, and adulthood; particularly, it is a direct cause of several childhood morbidities, including hypertension, dyslipidaemia, and the accelerated onset of cardiovascular disease and type-2 diabetes [1]. The complications arising from obesity have increased the costs of national health insurance and medical expenses, indicating that abnormal body weight in school children is a critical public health issue.

The fundamental cause of obesity and overweight is an energy imbalance between calories consumed and calories expended. Globally, an increased intake of energy-dense foods high in sugar and fat, and an increase in physical inactivity stemming from children’s obsession with electronic products (televisions, computers, and mobile phones) have been observed [1,5]. Studies have demonstrated that the BMI of school children is related to their physical activity levels [6,7]. Moreover, most school children consume an inadequate amount of fiber, whereas sweet foods account for approximately 50% of their daily caloric intake [8]. The results of one body weight and healthy diet study conducted on 2208 elementary school children in Taiwan by Liou, et al. [9] indicated that the percentage of overweight or obese students with a healthy dietary intake is significantly lower than in students without a healthy dietary intake. Furthermore, sedentary behaviors (obsession with the Internet and electronic products), which leads to inadequate physical activity levels, is also a major cause of overweight and obesity in school children [10]. In addition, body shape perception has a lasting effect on the body weight of children and early adolescents [11], and bodily dissatisfaction may lead to the development of a subsequent eating disorder or obesity [12]. Research has indicated that weight misperception has a tendency for overweight or obese in children [13] and body shape has a lasting effect on the body weight of children by the age of 10 years [11].

The early detection of body weight problems in school children can help prevent chronic systemic diseases, cancer, and mortality, which may reduce the medical expenditures [1] caused by obesity; thus, a foundation for the healthy lifestyles of adults should be established. The Taiwan Ministry of Education has made several policies for healthy body weight, such as increased physical activities, healthy lifestyle models, and balanced diets to assist elementary students in maintaining healthy body weight [14]. Through literature review on factors leading to obesity of schoolchildren in Taiwan, most of the studies were found to investigate only one influencing factor on obesity, such as physical activity, diet habits, sedentary lifestyle, or body image [9,10,13]. Single-factor research does not assume a holistic view. As schoolchildren in southern Taiwan have the highest proportion of abnormal BMI [3], it is crucial to explore the multiple impact factors on obesity of schoolchildren from an overall perspective. The purpose of this study is to explore multiple factors which including physical activity levels, sedentary behaviors, dietary habits, and perceived body shape on body mass index of elementary students in southern Taiwan.

## 2. Materials and Methods

This study was approved by the Institutional Review Board of Chang Gung Hospital in southern Taiwan (approval number: 102-4399B). The participants comprised fifth-grade students, who were recruited from 129 elementary schools in a southern Taiwan county that had the highest rates of childhood obesity countrywide. Both the selected children and their parents were oriented on the objectives and procedures of this study, following which they provided written informed consent for participation.

### 2.1. Design and Sampling

A cross-sectional study was designed and conducted on fifth-grade students from 129 elementary schools in southern Taiwan. Participants were required to meet the following inclusion criteria: (1) be a fifth-grade student in elementary school and (2) voluntarily agree to participate in the study. Individuals requiring special education classes or those with disabilities were excluded from the study. From September 2013 to May 2014, this comprehensive survey was conducted on all the 5th graders of 129 primary schools in the southern part of Taiwan. 3442 schoolchildren who met the criteria were recruited in this study, and a final total of 3251 (1628 male and 1623 female) schoolchildren completed the program, with a 94.5% response rate. One hundred and ninety-one (5.5%) participants were excluded due to missing responses more than five questions or evidence showing the participants had not taken the responses seriously.

### 2.2. Instruments

#### Anthropometric Measurements

The height and weight of the participants, barefooted and in light clothing, were measured during the first semester in September 2013. All measurements were performed by trained teachers, according to standard operating procedures. Teachers calculated BMIs by dividing the weight by the square of the participants’ height (BMI = weight (kg)/height (m)^2^). The definition of childhood and adolescent obesity was based on the BMI values suggested by the Ministry of Health and Welfare in Taiwan for normal weight (BMI range: 14.8–20.7), underweight (BMI < 14.8), overweight (BMI ≥ 20.7), and obese (BMI ≥ 23.2) in 11-year-old boys, and for normal weight (BMI range: 14.7–20.5), underweight (BMI < 14.7), overweight (BMI ≥ 20.5), and obese (BMI ≥ 22.7) in 11-year-old girls [15].

Demographic questionnaire. This researcher-developed instrument was used to gather information related to school children’s demographic characteristics, including sex, BMI, and residential area.

Healthy body weight. This questionnaire, established by Liou [16], was specifically designed for 10–12-year-old Taiwanese school children to assess physical activity levels, diet habit, sedentary lifestyle, and body shape perception. It comprises 30 questions to assess the following seven aspects: (1) physical activity; (2) sleep; (3) sedentary behavior; (4) dietary habit; (5) confidence; (6) knowledge; and (7) perceived body shape. In Liou’s studies [17,18] demonstrated that this scale had well content validity index (CVI) and acceptable test/retest reliability. With consent from the author, a portion of the questionnaire contents were extracted for use: physical activity levels; sedentary behaviors (time spent watching television, playing video games, or using the computer); dietary habits; and perceived body shape.

A self-administered physical activity questions were obtained, and participants completed the questions according to the instructions of trained school teachers. The content validity index of this scale was higher than 0.9. The repeatable data of the scale produced Spearman’s ρ clustered around 0.67 [17]. Physical activities levels included seven items were classified into moderate (e.g., walking, gymnastics, dancing, and free play) or vigorous (e.g., jogging/running, swimming, and ball games) physical activities, and the participants were asked (e.g., “For how many minutes do you engage in moderate physical activities daily?” and “For how many minutes do you engage in vigorous physical activities daily?” Possible answers were not sure, less than 30 min, 31–60 min, and more than 60 min. The participants were also asked “How much time do you engage in walking daily?”). The possible answers were not sure, <30 min, 31–60 min, 61–120 min, and >120 min.

The scale of sedentary behaviors contains four items. This scale was evaluated by experts and had a content validity index of 0.99. The test-retest reliability in a 2-week interval was 0.84 by intra-class correlation coefficient [18]. The self-administered questions about sedentary behaviors (time spent watching television, playing video games or using the computer) were “How many times weekly do you watch television, play video games, or use the computer?” (none, 1–2 times, 3–4 times, 5–6 times, and >7 times) “How many hours do you spend daily watching television” (none, <1 h, 1–2 h, 2–3 h, >3 h), and “How many hours do you play video games, or use the computer during holiday?” (None, <1 h, 1–2 h, 2–3 h, >3 h) etc.

The scale of dietary habits contains five items. This scale was evaluated by experts and had a content validity index of 0.89. The test-retest reliability in a 2-week interval was 0.84 by intra-class correlation coefficient. The correlation with a 3-day dietary log ranged from 0.74 to 0.77 [18]. The self-administered questions on dietary habits were obtained, and participants completed the questions according to the instruction of dieticians. The participants were asked “Do you add vegetable or meat gravy to your rice?” (never, seldom, sometimes, often, and always), “How much amount of vegetables do you eat per day?” (none, less than half a fist, half a fist, one fist, and more than one fist), and “How many times do you eat wholegrain meals in a week?” (none, 1–3 times, 4–6 times, and >7 times) etc. 

The scale of perceived body shape contains seven items. The self-administered questions on perceived body shape were “What is your weight category?” (normal weight, slightly underweight, underweight, lightly obese, and obese) and “How satisfied are you with your body shape?” (very satisfied, happy, unsatisfied, and very unsatisfied), etc. The participants were also asked to recall and record their activities for researchers to further determine the frequency and duration of physical activities, sedentary behaviors, and dietary habits.

### 2.3. Statistical Analysis

The SPSS 17.0 software package (SPSS Inc., Chicago, IL, USA) was used for statistical analysis. Descriptive statistics (frequency distributions and percentages) were used to describe the participant demographic characteristics. Chi-squared test was performed to examine the differences in sex, residential area, physical activity levels, watching television/playing video games or using the computer, dietary habits, and body shape among various BMI categories. Multiple logistic regression was conducted to analyze the influence of sex, residential area, physical activity levels, sedentary behaviors, dietary habits, and perceived body shape on BMI.

## 3. Results

Table 1 summarizes the demographic data of the participants. This study selected 3251 fifth-grade students from 129 elementary schools. The average age of the participants was 10.5 ± 0.5 years, and 1628 (50.1%) were male and 1623 (49.9%) were female. The BMI of the male students ranged between 10.4 and 38.65 kg/cm, with an average value of 19.69 kg/cm; the BMI of the female students ranged between 10.63 and 36.39 kg/cm, with an average value of 18.70 kg/cm. According to the definition of childhood and adolescent obesity released by Health Promotion of the Ministry of Health and Welfare, the BMI of the participants was classified into underweight (*n* = 651; 20.0%), normal weight (*n* = 1595; 49.1%), overweight (*n* = 450; 13.8%), and obese (*n* = 555; 17.1%) groups.

Table 2 shows the variables and BMI categories. Chi-squared tests were conducted to determine statistical significance. First, the results revealed a statistical significance (χ^2^ = 44.6; *p* < 0.001) in the relationship between BMI and sex. Specifically, the adjusted residual (AR) values revealed that the percentage of girls with normal weight BMIs was significantly higher than that of boys, whereas the percentage of girls with overweight and obese BMIs was significantly lower than that of boys. By contrast, no statistical significance (χ^2^ = 4.8; *p* = 0.56) was found between BMI and residential area.

However, the AR values revealed that the percentage of participants with normal BMIs who lived in mountainous areas was higher than that of participants with normal BMIs who lived in coastal regions or on the plains. The results also indicated statistical significance between BMI and engage in vigorous physical activities daily (χ^2^ = 28.9; *p* < 0.001), engage in moderate physical activities daily (χ^2^ = 22.0; *p* < 0.01), and time spent walking (χ^2^ = 28.1; *p* < 0.05). The AR values revealed that the percentage of normal body weight among participants who engaged in 30–61 min of walking daily was higher than that among participants who engaged in <30 min or >60 min of walking daily. The results demonstrated that statistical significance between BMI and spend watching television daily (χ^2^ = 62.5; *p* < 0.001), and engage in video games or computer usage every holiday (χ^2^ = 55.6; *p* < 0.001).

Moreover, statistical significance was noted between BMI and the consumption of vegetable or meat gravy on rice (χ^2^ = 44.5; *p* < 0.001). The AR values revealed that the percentage of normal body weight among participants who seldom add vegetable or meat gravy to their rice was significantly higher than that among participants who always added vegetable or meat gravy to their rice. Conversely, no significance was found between BMI and the number of whole grain meals consumed per week (χ^2^ = 9.5; *p* = 0.38). The AR values here revealed that the percentage of obese participants who did not consume whole grain meals was higher than that of participants in other BMI categories. Statistical significance was also observed between body shape and BMI (χ^2^ = 489.8; *p* < 0.001). The AR values revealed that the percentage of participants with normal BMIs who were very satisfied or happy with their body shape was significantly higher than that of participants who were very unsatisfied or unsatisfied with their body shape. 

Table 3 shows the results of multiple logistic regression analysis on sex, physical activities, watching television, playing video games or using the computer, body shape and the participants’ BMI. The risk of an abnormal BMI in boys was higher than that in girls (odds ratio (OR), 1.369; 95% confidence interval (CI), 1.172–1.599). 

Additionally, the risk of an abnormal BMI in participants who engaged in <30 min of daily vigorous exercises was higher than that in participants who engaged in 31–60 min of daily vigorous exercises (OR, 0.716; 95% CI, 0.529–0.968). Participants who engaged in <30 min of moderate physical activities daily also had a higher risk of having an abnormal BMI than that those who engaged in 31–60 min moderate physical activities daily (OR, 0.637; 95% CI, 0.428–0.949). Furthermore, participants who watched 2–3 h of television daily were more likely than those who did not watch television daily to have an abnormal BMI (OR, 1.510; 95% CI, 1.025–2.226). Similarly, the risk of an abnormal BMI was higher in participants who engaged in 1 h of video games or computer usage during every holiday than it was in those who did not engage in video games or computer usage (OR, 0.747; 95% CI, 0.596–0.935). The risk of abnormal BMI in participants who engaged in at least 2–3 h of video games or computer usage every week was higher than that in participants who did not engage in video games or computer usage during holiday (OR, 0.648; 95% CI, 0.482–0.871). The risk of an abnormal BMI in participants who were very unsatisfied with their body shape was higher than that in participants who were very satisfied with their body shape (OR, 3.839; 95% CI, 2.764–5.332). The risk of an abnormal BMI in participants who were unsatisfied with their body shape was higher than that in participants who were very satisfied with their body shape (OR, 2.257; 95% CI, 1.667–3.055). Finally, the risk of an abnormal BMI in participants who were happy with their body shape was higher than that in participants who were very satisfied with their body shape (OR, 1.385; 95% CI, 1.130–1.697).

## 4. Discussion

The results of this study demonstrated that the prevalence of overweight and obesity among school children of both sexes (boys: 36.3%; girls: 25.5%) from the southern county of Taiwan was high than the prevalence of these conditions reported among a similar group of school children (boys: 34.0%; girls: 25.0%) in 2011 [19] and significant higher than that found in the national survey in Taiwan 2012 (boys: 31.70%; girls: 25.1%) [20]. Given the combined high prevalence of overweight and obesity among southern Taiwanese youth, especially boys, we thus argue that this problem requires immediate health promotion intervention.

Our results indicated that the participants who did not engage in daily vigorous or moderate exercises for at least 31–60 min were more prone to abnormal BMIs than were their counterparts. Notably, 1530 (47.1%) participants engaged in an average that comprised 30 min of vigorous physical activities daily, and 1550 (47.7%) participants engaged in an average of 30 min of moderate physical activities daily; this was far from the 60 min of daily moderate-to-vigorous physical activity recommended by the WHO [21]. Physical activity, including physical level and actual physical performance, can decrease BMI [22]. Additionally, intervention programs, including programs that target physical activity and health education for obese youth, have been shown to significantly decrease BMI and result in an improvement in physical ability among their participants [22,23].

Regarding the residential areas examined in this study, the coastal and plain regions were classified as urban areas, whereas the mountain areas were classified as rural areas. The prevalence of overweight and obesity appears to be increasing among the youth living in inner cities that are undergoing rapid economic growth. This may be a result of the development of convenient transportation, a lack of adequate space and green land for exercising, and the easily accessible high-sugar content beverages and high-energy fast-food present in urban areas. By contrast, children in rural areas are more likely to walk to school, have more open spaces to engage in physical activity, and eat local fresh healthy food [24]; thus, the children in urban areas are more likely to become obese than are children in rural areas. We identified a positive correlation between the average time spent walking every day and normal body weight; this finding is consistent with research by Jiang et al. [25], who analyzed Chinese children aged 8–15 years and reported higher obesity rates for urban than for rural areas. Moreover, a walking program is beneficial at reducing the BMIs of school children [26]. Designing an urban environment that promotes convenient and secure open spaces for physical activities may increase the moderate-to-vigorous physical activity levels of children [27] and reduce the prevalence of childhood obesity.

A sedentary lifestyle is also a key risk factor for childhood obesity [5]. The present study demonstrated that participants who engaged in >60 min of television, video games, or computer usage were more prone to abnormal BMIs than those who did not engage in excessive television, video games, and computer usage. Similarly, Liou et al. [18] investigated the relevance between obesity and the sedentary lifestyles of 8640 Taiwanese students aged 13–16 years. They reported that watching television for ≥120 min/day on weekdays was a significant risk factor for obesity among both girls and boys. Few longitudinal studies have indicated children who watch television have a higher risk of becoming overweight and obese adolescents [28,29]. Interventions targeted at helping Taiwanese parents developing healthy television-viewing and computer-game-playing practices in their young children are clearly warranted.

The high consumption of sugar and oils is associated with an increased risk of obesity. The present study demonstrated that the percentage of normal body weight among participants who did not add vegetable or meat gravy to their rice was significantly higher than that among participants who occasionally or always added vegetable or meat gravy to their rice. Previous studies have echoed this idea that sugar and oil intake is associated with an increased risk for overweight and obesity [30,31]. One longitudinal study also investigated childhood dietary patterns and their association with childhood overweight/obesity, and reported that promoting a diet rich in whole meal cereals may counteract overweight and obesity in children [32].

Family is one of the key factors affecting the dietary habits of children, because parents play a pivotal role in helping their children establish healthy dietary habits and preventing the occurrence of obesity. Hence, preventative measures for childhood obesity should consider family dietary habits [33,34]. To control BMI, Esfarjani et al. [35] conducted a family-based intervention study in an attempt to decrease oil and sugar consumption among 156 obese children, and discovered that the family diet-style program had desirable effects. Considering the high-sugar and -oil cooking methods that are part of Taiwan’s exquisite culinary culture, parents are encouraged to provide a positive example for their children by choosing foods with high dietary fibers and adopting low-sugar and -oil culinary methods [36], which thus encourage their children to adopt similarly healthy dietary patterns and develop dietary habits for healthy body weight.

Body shape is also positively associated with BMIs in school children [37]. Findings of the present study indicated that participants who were satisfied with their body shape were more likely to have a normal BMI. Similarly, Chen et al. [38] estimated the body shape of 883 Taiwanese early adolescents and its relationship with weight status, and concluded that body shape dissatisfaction is related to overweight. A poorly perceived body shape during childhood is associated with long-term problems with obesity, eating disorders, and negative body image [12]. For decades, the media and commercial advertisements have stressed the concept of ‘slim is beauty’ to the entire world, and school children have become increasingly preoccupied with their body weight because of this socio-cultural norm that is reinforced by media messages. To avoid negative consequences, incorporating the components of perceived body shape in obesity intervention programs at schools should be a primary responsibility of public healthcare providers [39]. We suggest that a school-based healthy body weight program comprising information about healthy body weight, body image, and media literacy could prevent abnormal body weight and improve body satisfaction among school children entering puberty.

### Limitations

The results of this study not only make the government and schools more aware of the overweight or obesity factors that are affecting schoolchildren in southern Taiwan, but they also propose an effective program to rectify the obesity factors, resulting in an improvement on the obesity rate for both male and female in southern Taiwan. Nevertheless, the research design and sample in this study had some limitations. This study only examined fifth-grade school children in southern Taiwan, and thus the findings cannot necessarily be used to deduce the conditions of school children in other regions of Taiwan. Future researchers may expand the scope to other counties and cities to offer a more representative image of Taiwanese conditions. Additionally, because this was a cross-sectional study, the information collected in the questionnaires only definitively reflected the conditions when the survey was conducted; causality cannot be determined. We suggest that future researchers conduct a longitudinal study or introduce experimental research on physical activities, dieting, and lifestyles to ensure that school children develop healthy exercising and dieting habits and lifestyles during elementary school, thereby achieving the objectives of health promotion. Finally, because the survey was conducted in the form of retrospective self-assessment questionnaires, inaccuracies in the results may be present because of young-age-related limitations in the participants (e.g., cognition, seriousness of questionnaire completion, and memory factors). Future researchers may elect to train pilot instructors to offer explanations while the children are completing the questionnaire to increase the accuracy of the survey.

## 5. Conclusions

Female school children and school children who engage in 31–60 min of moderate-to-vigorous physical activities daily, do not engage in television watching after school, have healthy diets, do not add gravy or soup to their rice, and are satisfied with their body shape are overall less likely to have abnormal BMIs. Thus, education policies should increase the amount of school time allocated to physical activities, educate school children on healthy body weight related information, encourage parents to arrange outdoor activities, and establish healthy dietary habits to ensure that families and schools can cooperate in assisting children to maintain a healthy body weight. Moreover, the body shape satisfaction should draw special attention from school teachers. They can use the study results to guide schoolchildren in their early adolescence to be more satisfactory of their body shape and more self-appreciative. Thus, schoolchildren will enhance self-confidence and interpersonal relationships, and their physical, emotional, mental and spiritual development will not be affected only because of abnormal BMI leading to inferiority, isolation and other negative personality traits. The study results have once again verified that gender, physical activity levels, sedentary behaviors, diet habits, and body shape satisfaction are high risk factors for obesity. This result deserves the concern and attention of the worldwide.

## Figures and Tables

**Table 1:** Demographic data (*n* = 3251).

Variables	Mean (SD, Range)	*n*	%
Sex			
Male		1628	50.1
Female		1623	49.9
Residential area			
Mountain regions		874	26.9
Coastal regions		986	30.3
Plain regions		1391	42.8
BMI			
Male	19.69 (4.20, 10.4–38.65)	1628	
Underweight		287	17.6
Normal weight		751	46.1
Overweight		250	15.4
Obese		340	20.9
Female	18.70 (3.74, 10.63–36.39)	1623	
Underweight		364	22.5
Normal weight		844	52.0
Overweight		200	12.3
Obese		215	13.2

**Table 2:** Variables and BMI categories (*n* = 3251).

Variables	BMI												χ^2^
	Underweight			Normal Weight			Overweight			Obese			
	n/%/AR												
Sex													44.6 ***
Male	287	17.6%	−3.4	751	46.1%	–3.1	250	15.4%	4.4	340	20.9%	4.0	
Female	364	22.4%	3.4	844	52.0%	3.1	200	12.3%	–4.4	215	13.2%	–4.0	
Residential area													4.8
Mountainous Regions	185	21.2%	1.0	456	52.2%	0.6	101	11.6%	–1.3	132	15.1%	–0.7	
Costal regions	193	19.6%	–0.4	495	50.2%	–0.9	142	14.4%	1.8	156	15.8%	0.0	
Plain regions	273	19.6%	–0.5	718	51.6%	0.3	173	12.4%	–0.5	227	16.3%	0.6	
Engage in vigorous physical activities daily													28.9 ***
<30 min	322	21.0%	1.3	768	50.2%	–1.2	191	12.5%	–0.4	249	16.3%	0.6	
31–60 min	139	19.9%	–0.1	396	56.7%	3.2	77	11.0%	–1.6	87	12.4%	–2.8	
>60 min	65	15.4%	–2.6	234	55.5%	1.8	52	12.3%	–0.3	71	16.8%	0.6	
Not sure	125	21.0%	0.6	269	45.2%	–3.3	94	15.8%	2.5	107	18.0%	1.6	
Engage in moderate physical activities daily													22.0 **
<30 min	327	21.1%	1.5	783	50.5%	–0.9	193	12.5%	–0.6	247	15.9%	0.1	
31–60 min	144	19.0%	–0.8	413	54.6%	2.1	91	12.0%	–0.7	108	14.3%	–1.3	
>60 min	59	15.5%	–2.3	218	57.4%	2.5	44	11.6%	–0.8	59	15.5%	–0.2	
Not sure	120	21.5%	0.9	251	44.9%	–3.3	88	15.7%	2.3	100	17.9%	1.5	
Walking													
Engage in walking daily													28.1 *
<30 min	360	21.6%	2.3	851	51.1%	–0.2	211	12.7%	–0.3	242	14.5%	–2.0	
31–60 min	101	17.1%	–2.0	323	54.6%	1.7	73	12.3%	–0.4	95	16.0%	0.2	
60–120 min	32	21.6%	0.5	77	52.0%	0.2	21	14.2%	0.5	18	12.2%	–1.2	
>120 min	56	19.0%	–0.5	156	52.9%	0.6	23	7.8%	–2.7	60	20.3%	2.2	
Not sure	101	18.6%	–0.9	257	47.2%	–2.1	88	16.2%	2.6	98	18.0%	1.5	
Spend watching television daily													62.5 ***
>3 h	99	19.0%	–0.6	234	45.0%	–3.1	71	13.7%	0.6	116	22.3%	4.4	
2–3 h	72	15.9%	–2.4	209	46.0%	–2.4	70	15.4%	1.8	103	22.7%	4.3	
1–2 h	176	20.1%	0.0	466	53.1%	1.3	116	13.2%	0.4	119	13.6%	–2.2	
<1 h	244	21.1%	1.2	623	53.9%	2.2	135	11.7%	–1.4	153	13.2%	–3.0	
None	59	24.4%	1.8	135	55.8%	1.4	24	9.9%	–1.4	24	9.9%	–2.6	
Playing video games or using the computer during holiday													55.6 ***
>3 h	101	18.3%	–1.1	242	43.9%	–3.8	85	15.4%	2.0	123	22.3%	4.6	
2–3 h	64	15.8%	–2.2	221	54.6%	1.4	52	12.8%	0.0	68	16.8%	0.6	
1–2 h	137	19.3%	–0.5	357	50.3%	–0.6	108	15.2%	2.2	108	15.2%	–0.5	
<1 h	200	20.4%	0.4	551	56.3%	3.7	106	10.8%	–2.2	122	12.5%	–3.5	
None	147	24.4%	3.0	297	49.3%	–1.1	65	10.8%	–1.7	94	15.6%	–0.2	
Add vegetable or meat gravy to rice													44.5 ***
Always	39	16.2%	–1.5	97	40.2%	–3.6	45	18.7%	2.8	60	24.9%	4.0	
Often	77	19.4%	–0.3	197	49.6%	–0.7	61	15.4%	1.6	62	15.6%	–0.1	
Sometimes	232	21.0%	1.0	569	51.5%	0.1	132	11.9%	–1.1	172	15.6%	–0.3	
Seldom	211	18.3%	–1.8	624	54.0%	2.2	145	12.5%	–0.3	176	15.2%	–0.7	
Never	90	25.7%	2.8	182	52.0%	0.2	33	9.4%	–2.0	45	12.9%	–1.6	
Eat whole grain meals in a week													9.5
None	76	19.0%	–0.5	199	49.8%	–0.7	48	12.0%	–0.5	77	19.3%	2.0	
1–3 times	241	18.7%	–1.4	672	52.3%	0.8	177	13.8%	1.3	196	15.2%	–0.8	
4–6 times	150	20.0%	0.0	394	52.5%	0.7	93	12.4%	–0.4	114	15.2%	–0.6	
>7 times	182	22.4%	2.0	404	49.8%	–1.1	98	12.1%	–0.7	128	15.8%	–0.1	
Body shape													489.8 ***
Very unsatisfied	47	16.4%	–3.5	189	66.1%	–4.5	115	40.4%	4.4	218	77.0%	7.5	
Unsatisfied	262	18.4%	–2.0	729	51.3%	–0.2	200	14.1%	2.1	231	16.2%	0.5	
Happy	140	25.3%	3.4	344	62.1%	5.5	45	8.1%	–3.6	25	4.5%	–8.0	
Very satisfied	187	29.2%	6.5	377	58.9%	4.2	45	7.0%	–4.8	31	4.8%	–8.5	

* *p* < 0.05; ** *p* < 0.01; *** *p* < 0.001.

**Table 3:** Logistic regression analyzed sex, physical activities, sedentary behaviors, body shape and BMI (*n* = 3251).

Variables	β	SD	*p*	OR	95% CI
Sex					
Male	0.314	0.79	0.00	1.369	1.172–1.599
Female	Reference				
Engage in vigorous physical activities daily					
31–60 min	−0.335	0.154	0.03	0.716	0.529–0.968
<30 min	Reference				
Engage in moderate physical activities daily					
31–60 min	−0.451	0.203	0.027	0.637	0.428–0.949
<30 min	Reference				
Spend watching television daily					
2–3 h	0.412	0.198	0.037	1.510	1.025–2.226
None	Reference				
Playing video games or using the computer during holiday					
2–3 h	−0.433	0.151	0.004	0.648	0.482–0.871
1 h	−0.292	0.115	0.011	0.747	0.596–0.935
None	Reference				
Body shape					
Very unsatisfied	1.345	0.168	0.000	3.839	2.764–5.332
Unsatisfied	0.814	0.155	0.000	2.257	1.667–3.055
Happy	0.326	0.104	0.002	1.385	1.130–1.697
Very satisfied	Reference				
